# Inscrutable games? Facial expressions predict economic behavior

**DOI:** 10.1186/1471-2202-12-S1-P281

**Published:** 2011-07-18

**Authors:** Filippo Rossi, Ian Fasel, Alan G Sanfey

**Affiliations:** 1 Department of Psychology, University of Arizona, Tucson, AZ 85721, USA; 2 Department of Computer Science, University of Arizona, Tucson, AZ 85721, USA; 3 Donders Institute for Brain, Cognition and Behaviour, Radboud University, Nijmegen, NL-6500 HB, Netherlands

## 

**Figure 1:**
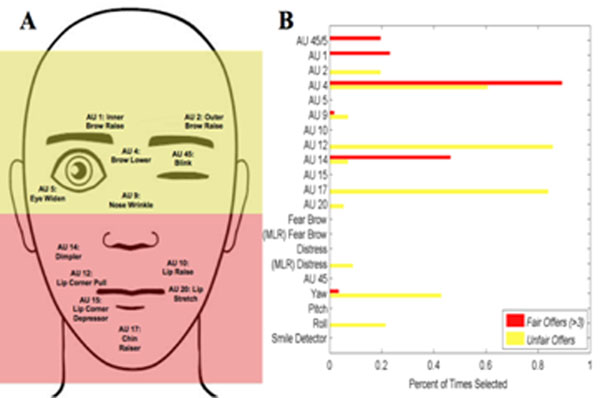
A. Action units (AUs) used in the analysis (image of the face created with Artnatomy [5]). B. Frequency with which a feature is selected as covariate in a logistic classifier, using increases in area under the ROC as inclusion criterion.

Neuroscientific and behavioral evidence shows that when subjects are engaged in simple economic games, they pay attention to the face of their opponents. Is this a good idea? Does the face of a decision-maker contain information about his strategy space? We tested this hypothesis by modeling facial expressions of subjects playing the Ultimatum Game. We recorded videos of 60 participants, and automatically extracted time-series of facial actions (12 action units [1], shown in Fig. 1A., as well as pitch, yaw, and roll of the head) using the real-time facial coding system of [2,3]. We then trained non-linear support vector machines (SVM) to predict the decision of the second player from a segment of video acquired after the offer was received and before the decision was entered (n = 376). To separate the dynamics of facial behavior into different temporal scales, the data was preprocessed with a bank of Gabor filters. With this method we achieved a between-subjects cross-validation accuracy of 0.66 (chance = 0.50) in predicting decisions. Because receiving an unfair offer in the Ultimatum Game is known to evoke a differential facial expression [4], we also trained a model which can capture non-linear relations between facial expressions, fairness and decisions. To do so, we labeled each instance as fair (offer > $3) or unfair, and then trained different classifiers to be ‘experts’ on either fair or unfair offers only. In this case, out-of-sample classification accuracy increased to 0.78. For both cases, we used a foreword selection procedure to identify the most predictive features (Fig.1B).

Abstract approaches that study social decision-making usually disregard the fact that choices are made in informationally rich environments. Instead, one important goal is to model different sources of information as well as the way in which they affect decisions. The current study suggests that one important source of information about strategic decision making behavior may be the face, since, given sensitive enough instruments, this information can be measured and quantified in real-time by a computer. This also suggests that real-time analysis of facial action codes may serve as a powerful new tool for understanding strategic decision making which can complement neuroimaging techniques such as EEG and fMRI.
